# The Putative RNA Helicase HELZ Promotes Cell Proliferation, Translation Initiation and Ribosomal Protein S6 Phosphorylation

**DOI:** 10.1371/journal.pone.0022107

**Published:** 2011-07-13

**Authors:** Philippe A. Hasgall, David Hoogewijs, Marius B. Faza, Vikram G. Panse, Roland H. Wenger, Gieri Camenisch

**Affiliations:** 1 Institute of Physiology and Zürich Center for Integrative Human Physiology ZIHP, University of Zürich UZH, Zürich, Switzerland; 2 Institute of Biochemistry, ETH Zürich, Zürich, Switzerland; Tulane University Health Sciences Center, United States of America

## Abstract

The hypoxia–inducible transcription factor (HIF) is a key component of the cellular adaptation mechanisms to hypoxic conditions. HIFα subunits are degraded by prolyl-4-hydroxylase domain (PHD) enzyme-dependent prolyl-4-hydroxylation of LxxLAP motifs that confer oxygen-dependent proteolytic degradation. Interestingly, only three non-HIFα proteins contain two conserved LxxLAP motifs, including the putative RNA helicase with a zinc finger domain HELZ. However, HELZ proteolytic regulation was found to be oxygen-independent, supporting the notion that a LxxLAP sequence motif alone is not sufficient for oxygen-dependent protein destruction. Since biochemical pathways involving RNA often require RNA helicases to modulate RNA structure and activity, we used luciferase reporter gene constructs and metabolic labeling to demonstrate that HELZ overexpression activates global protein translation whereas RNA-interference mediated HELZ suppression had the opposite effect. Although HELZ interacted with the poly(A)-binding protein (PABP) via its PAM2 motif, PABP was dispensable for HELZ function in protein translation. Importantly, downregulation of HELZ reduced translational initiation, resulting in the disassembly of polysomes, in a reduction of cell proliferation and hypophosphorylation of ribosomal protein S6.

## Introduction

RNA helicases use ATP to modulate the structure of RNA, thereby altering the biologic activity of the RNA molecule or regulating access by other proteins. Virtually all biochemical processes involving RNA, including transcription, splicing, transport, translation, decay and ribosome biogenesis, employ helicases [1], [2]. The putative RNA helicase with a zinc finger domain HELZ belongs to the Upf1-like family of the superfamily I class of helicases [3], [4]. Members of this family have previously been implicated in mRNA processing [5], [6]. Tagged-HELZ has been shown to co-immunoprecipitate with co-transfected histone methyltransferases Smyd2 and Smyd3 and a functional role as adaptor molecule to RNA polymerase II has been suggested [7], [8]. However, the function of HELZ, especially in RNA metabolism, remained incompletely understood.

We identified two conserved LxxLAP motifs in the HELZ protein sequence which are known to be hydroxylated within the α-subunit of hypoxia-inducible transcription factors (HIFs). Transcriptional activation of oxygen-regulated genes by heterodimeric HIFs is a crucial step in the adaptation of mammalian cells to low oxygen [9], [10]. HIFα subunits are constitutively expressed but the protein stability is regulated by oxygen-dependent hydroxylation of specific prolyl residues located within the LxxLAP sequence by members of the prolyl-4-hydroxylase domain (PHD) enzyme family. Prolyl-4-hydroxylation is necessary for the interaction with the von Hippel-Lindau tumor suppressor protein (pVHL) that mediates HIFα degradation [11], [12], [13], [14], [15]. We report here that HELZ is oxygen-independently regulated, confirming that the sole presence of even two conserved LxxLAP motifs does not allow the prediction of oxygen-dependent regulation of protein stability.

Protein synthesis is regulated at various levels, but the limiting step is translational initiation. During this step, the 40S small ribosomal subunit is recruited to the mRNA 5′ end, scans towards a start codon and starts polypeptide synthesis by assembling the complete ribosome [16], [17]. Control of translation is often achieved by modulation of eukaryotic initiation factors (eIFs). The 5′ cap mRNA structure is bound by eIF4E, while eIF4G interacts with the poly(A)-binding protein (PABP) that associates with the 3′ poly(A) tail of eukaryotic mRNAs. eIF4A is a DEAD box RNA helicase that resolves mRNA secondary structures and eIF4B harbors two RNA-binding domains interacting with mRNA and the 18S small ribosomal subunit [18]. Whereas translational initiation is suppressed by association of 4E-binding proteins to eIF4E, translation is activated following 40S ribosomal protein S6 kinase (S6K)-mediated phosphorylation of eIF4B that is then recruited to the initiation complex and enhances RNA helicase activity of eIF4A. Central in the regulation of the activity of these translation initiation factors is the mammalian target of rapamycin (mTOR) signal transduction pathway that integrates information on nutrients, energy, stress, hormones and mitogens with modulation of protein synthesis [19].

Here, we provide evidence that HELZ is involved in global protein translation. Mechanistically, HELZ downregulation resulted in S6 hypophosphorylation and dissociation of polysomes, suppressing cell proliferation.

## Results

### The putative RNA helicase HELZ contains two conserved LxxLAP motifs

A comprehensive *in silico* screen for proteins containing two LxxLAP motifs, like the HIFα subunits, revealed several candidate LxxLAP containing proteins in the human genome. To narrow down this list of proteins we further evaluated conservation across several species, which is generally considered suggestive for functionality of a sequence motif. Surprisingly, besides HIF-1α and HIF-2α only three additional such proteins were identified: HELZ, CCR4-NOT transcription complex (CNOT1) and faciogenital dysplasia protein (FGD1) (Supporting Table S1). Whereas CNOT1 is important for regulation of mRNA synthesis and decay [20], FGD1 encodes a guanine nucleotide exchange factor, activating the GTPase Cdc42 that is important for regulation of membrane trafficking [21]. However, none of these proteins have previously been reported to be oxygen-dependently regulated.

**Figure 1:**
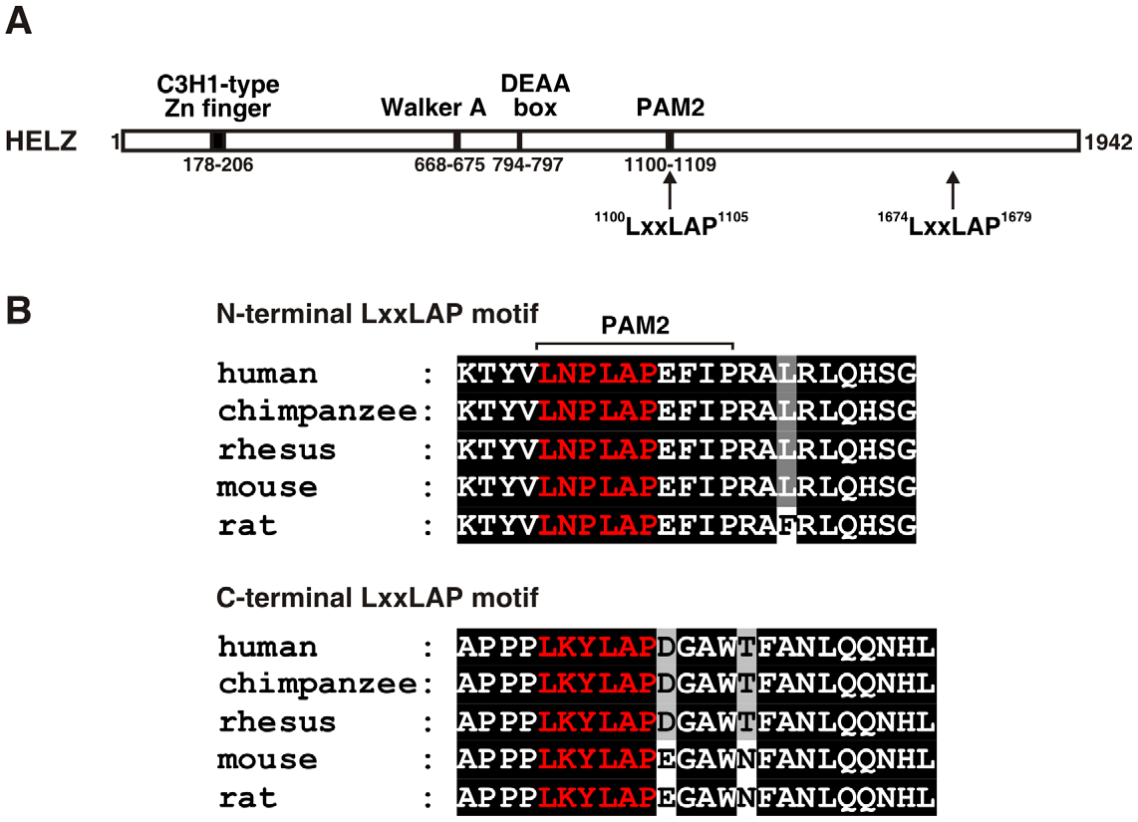
Human HELZ protein sequence analysis. (**A**) Schematic representation of the predicted human HELZ protein architecture. (**B**) Sequence alignment of the N-terminal LxxLAP motif that overlaps with the PAM2 motif (upper panel) and C-terminal LxxLAP motif (lower panel) of the indicated species. The LxxLAP motif is depicted in red and strictly conserved residues are shown as white letters on black background.

In our further studies, we concentrated on HELZ (accession number NP_055692.2), because coincidentally the same protein has been identified in a previously performed yeast two-hybrid screen for PHD2 interactors [22], suggesting that the two conserved LxxLAP motifs might be of functional relevance for interaction with the PHD oxygen sensors and probably oxygen-dependent regulation of HELZ protein stability and/or function. HELZ belongs to the superfamily I class of RNA helicases and contains a N-terminal C3H1-type zinc finger as well as typical helicase motifs including an ATP-binding domain (Walker A) important for helicase activity, a DEAA and a PAM2 motif, known to be required for interaction with the poly(A) binding protein (PABP) (Figure 1A). Both the N-terminal LxxLAP sequence that is part of the PAM2 motif, as well as the C-terminal LxxLAP motif are well conserved in mammals (Figure 1B upper and lower panel, respectively). The N-terminal LxxLAP motif is conserved to lower vertebrates like teleosts, whereas the C-terminal LxxLAP sequence is present only in placental mammals.

### HELZ protein abundance is oxygen-independently regulated

**Figure 2:**
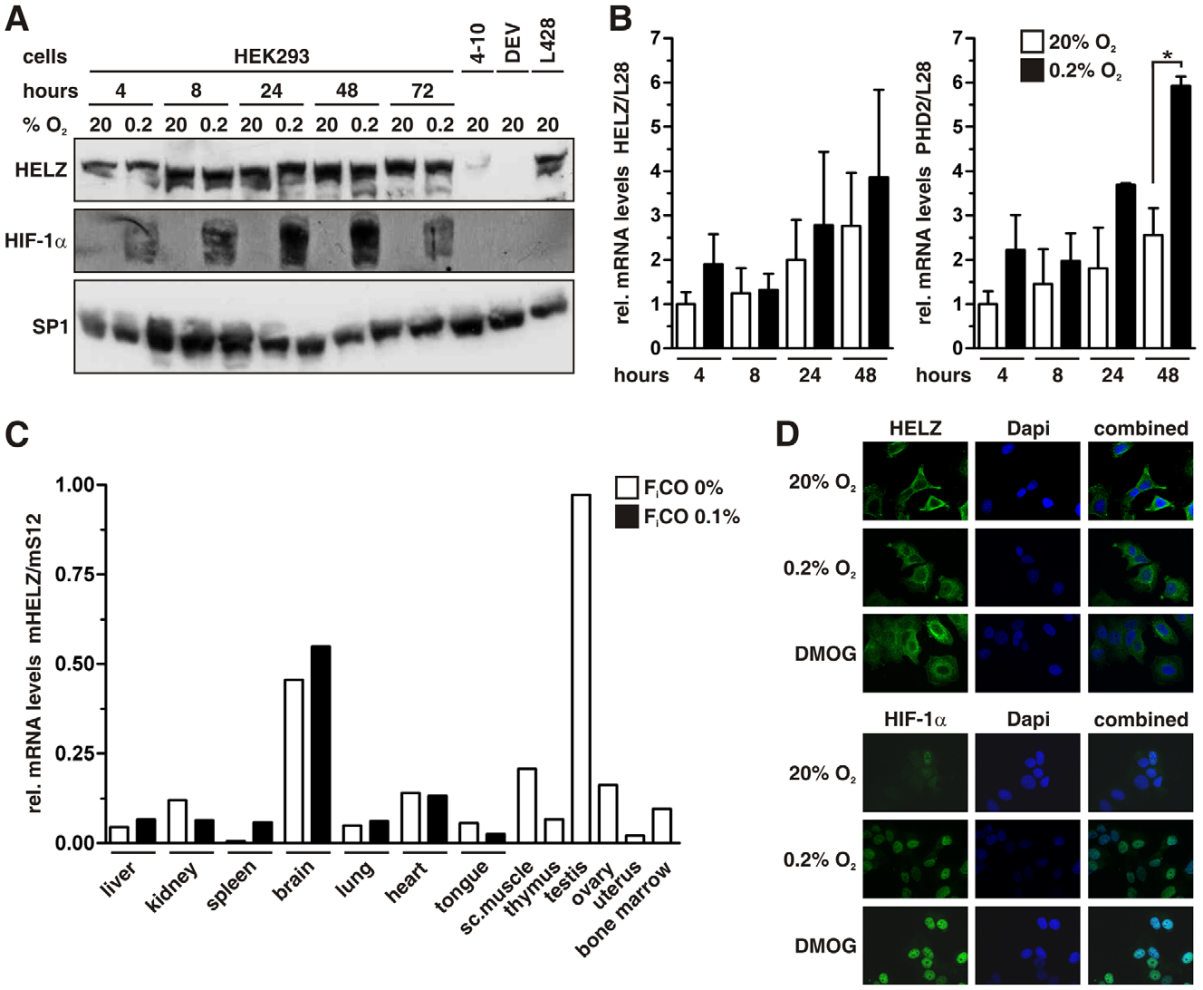
Oxygen-independent regulation of HELZ. (**A**) Immunoblot analysis of HEK293 cells cultured under normoxic or hypoxic conditions for the indicated time periods. The stable HELZ shRNA clone 4–10 and the Hodgkin's lymphoma-derived cell line DEV containing a homozygous deletion on chromosome 17 served as controls for reduced and absent HELZ protein levels, respectively. The Hodgkin's lymphoma derived cell line L428 served as positive control. (**B**) Transcript levels of HELZ were analyzed in HEK293 cells by RT-qPCR. Cells were exposed to hypoxia (0.2% O_2_) for up to 48 hours and PHD2 served as hypoxia-inducible control. (**C**) Total RNA was extracted from tissue samples of various organs from mice exposed to inspiratory air (0% F_i_CO) or 0.1% carbon monoxide (0.1% F_i_CO) for 4 hours and HELZ transcript levels were quantified by RT-qPCR and normalized to ribosomal protein S12 mRNA levels. (**D**) MCF-7 cells were cultured under 20% O_2_ or 0.2% O_2_ for 18 hours, fixed, and HELZ as well as HIF-1α protein visualized by indirect immunofluorescence.

To analyze whether the LxxLAP sequences of HELZ might have similar proteolysis regulating function as in HIFα subunits, we cultured human embryonic kidney HEK293 cells for different time periods under normoxic or hypoxic conditions and investigated HELZ protein abundance by immunoblotting (Figure 2A). However, whereas HIF-1α protein levels were hypoxically induced as expected, HELZ protein expression was not significantly affected and variations are probably due to difficulties in detecting the predicted 219 kDa protein by immunoblotting. Also the pan-PHD inhibitor dimethyloxalylglycine (DMOG) did not affect HELZ protein levels (data not shown). Anti-HELZ antibody specificity was verified by RNA interference-mediated HELZ knock-down in HEK293 cells (stable clone 4–10) as well as by using the Hodgkin's lymphoma-derived cell line DEV that contains a 3-megabase homozygous deletion at 17q24.1–24.2, which includes, amongst others, HELZ [23]. The cell line L428 is also derived from an Hodgkin's lymphoma, but wild-type for HELZ, and served as positive control (Figure 2A).

HELZ mRNA levels were determined by RT-qPCR and although showing some variations were not significantly regulated under hypoxic conditions (Figure 2B, left panel). Hypoxic PHD2 mRNA induction served as positive control (Figure 2B, right panel). Organ-specific expression of HELZ *in vivo* has not been reported so far. In the mouse, HELZ mRNA levels varied between different tissues with highest levels in testis and brain and lowest in spleen and uterus (Figure 2C). Mice treated for 4 hours with an inspiratory gas mixture containing 0.1% carbon monoxide, resulting in acute tissue hypoxia, displayed no significant variations in HELZ mRNA levels, whereas other HIF target genes have been found to be strongly induced in these tissue samples [24], [25]. Finally, HELZ subcellular localization was analyzed by confocal immunofluorescence microscopy and remained mainly cytoplasmatic with enhanced perinuclear staining, independent of oxygenation and PHD function (Figure 2D, upper panel). On the other hand, HIF-1α was almost undetectable under normoxic conditions and accumulated in the nucleus under hypoxic and PHD inhibiting conditions (Figure 2D, lower panel).

It has been shown that the HELZ interactor SMYD3 changes subcellular localization during cell cycle progression and several RNA helicases have been reported to shuttle between nucleus and cytoplasm [8], [26], [27] Thus, we synchronized the cell cycle of the human hepatoma cell line HuH-7 and examined HELZ subcellular localization during cell cycle progression by indirect immunofluorescence (Supporting Figure S1). However, HELZ subcellular localization was not altered during cell cycle progression, remaining mainly in the cytoplasm. In summary, these data suggest that the two LxxLAP motifs of HELZ might have either oxygen-independent functions, or oxygen-dependent functions that are not involved in proteolytic regulation like in the case of HIFα subunits, or no function at all. However, PHD-dependent modification of HELZ might still regulate HELZ function(s) other than protein abundance.

### HELZ interacts with the poly(A)-binding protein via its PAM2 motif

**Figure 3:**
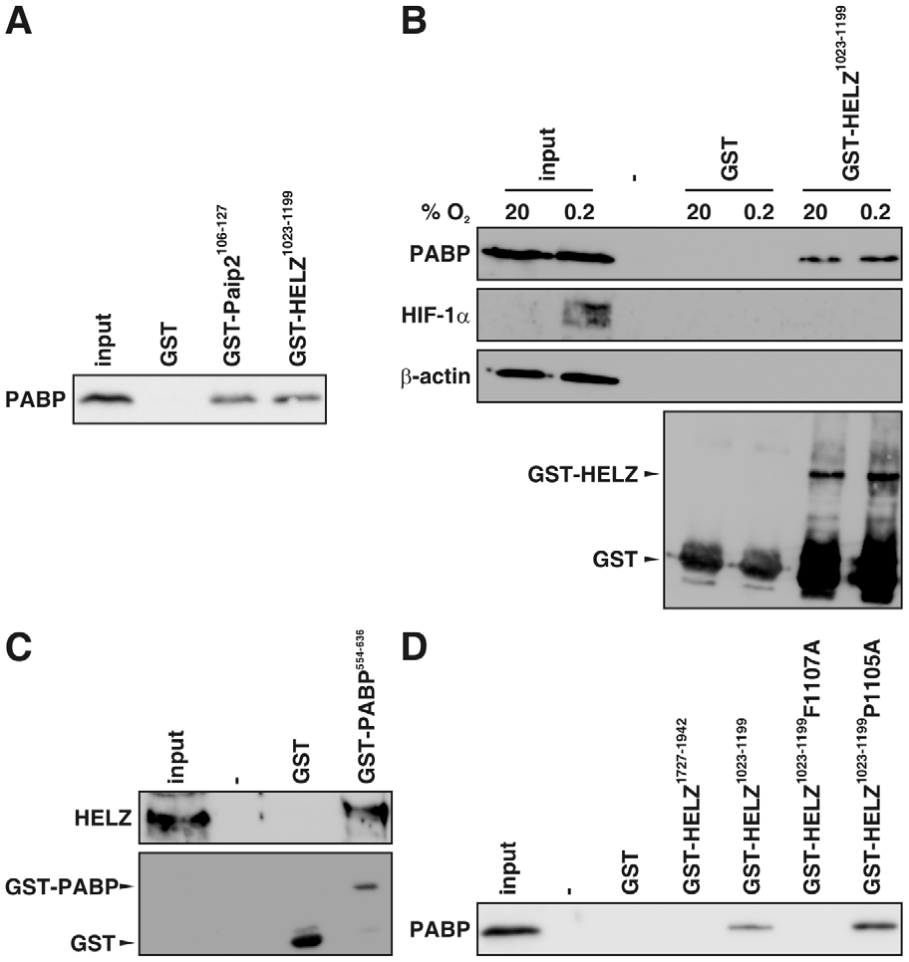
HELZ interacts with the poly(A)-binding protein (PABP). GST pull-down using glutathione-sepharose beads was conducted by incubating crude HeLa cell lysates with recombinant GST, GST-Paip2^106–127^ and GST-HELZ^1023–1199^ (**A**), GST and the indicated GST-HELZ fragments (**D**), or crude HEK293 cell lysates with GST and GST-PABP^554–636^ (**C**). Hypoxic HeLa cell lysates were incubated with GST or GST-HELZ^1023–1199^ (**B**). Eluates were subjected to SDS-PAGE and immunoblotting using the indicated antibodies.

The 3′ poly(A) tail of eukaryotic mRNAs is bound by PABP that interacts with eIF4G, a subunit of the multiprotein initiation complex that binds to the 5′ cap structure thereby regulating the initiation of translation. The PAM2 motif has originally been identified in the PABP interacting proteins Paip1 and Paip2 which regulate the activity of PABP [28]. The PAM2 motif is highly conserved in HELZ and overlaps with the N-terminal LxxLAP motif (Figure 1B). Therefore, we analyzed whether HELZ is able to interact with PABP by performing pull-down assays using HeLa cell lysates and GST-tagged HELZ fragments. As shown in Figure 3A, the PAM2 motif-containing GST-HELZ^1023–1199^ fragment interacted with PABP. GST-Paip2^106–127^ and GST alone served as positive and negative controls, respectively. Equal input was controlled by Coomassie staining (data not shown). PABP from hypoxic HeLa cell extracts also associated with recombinant GST-HELZ^1023–1199^ (Figure 3B) suggesting that the PABP:HELZ interaction was proline hydroxylation independent. Vice-versa, GST-PABP^554–636^ incubated with HEK293 cell lysates interacted with endogenous HELZ (Figure 3C).

The phenylalanine residue in the PAM2 motif of Paip1 and Paip2 is known to be critical for PABP binding [29]. Mutation of the corresponding phenylalanine in HELZ (F1107A) abrogated association with PABP, whereas mutation of the proline residue within the N-terminal LxxLAP sequence (P1105A) had no effect (Figure 3D). GST and GST-HELZ^1727–1942^ served as negative controls. Furthermore, the HELZ PAM2 motif overlaps with the eIF4E-binding signature YxxxxLΦ (x designates any amino acid and Φ a hydrophobic residue) [30] and we therefore tested, whether HELZ can interact with eIF4E. Wild-type PAM2 motif-containing HELZ fragments also interacted with eIF4E, but probably indirectly via binding to PABP since the interaction was abrogated when the phenylalanine residue critical for PABP binding was mutated (F1107A) (Supporting Figure S2).

### HELZ is involved in global protein translation

**Figure 4:**
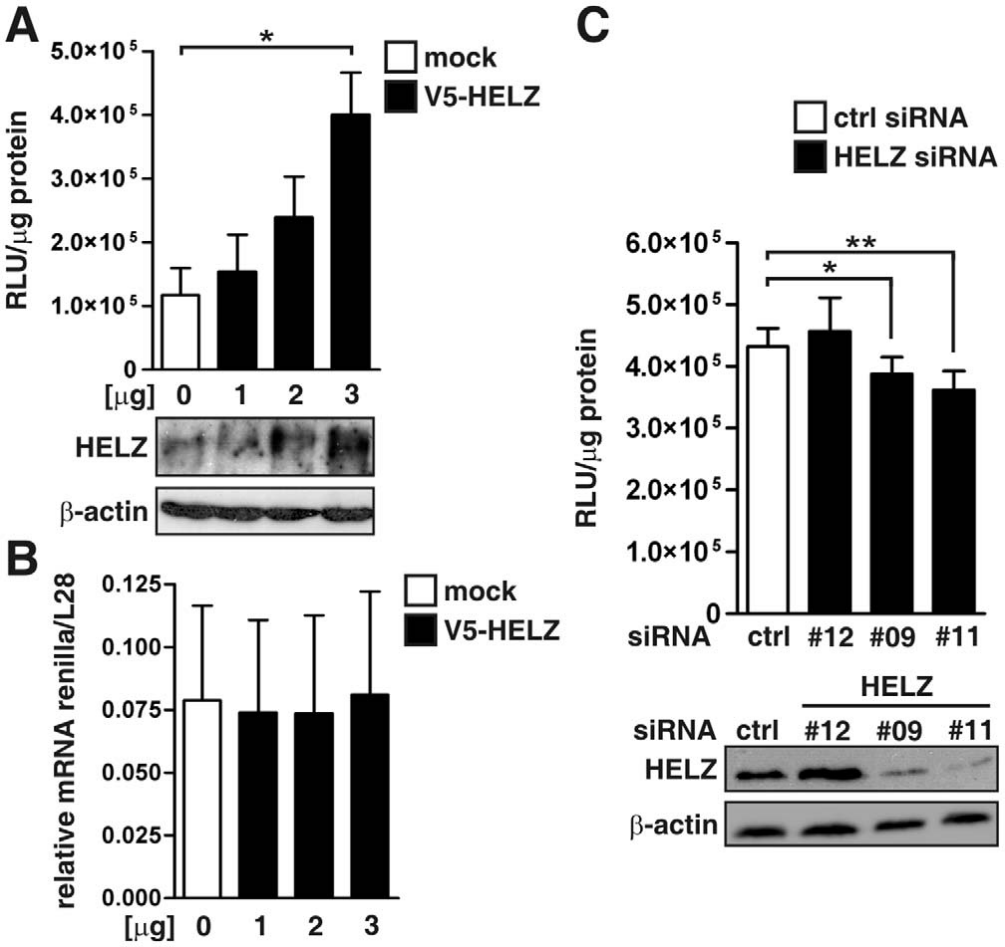
Translation of a luciferase reporter gene plasmid is regulated by HELZ. (**A**) HeLa cells were transiently co-transfected with the indicated amounts of HELZ or a control expression vector together with pRL-SV40 renilla luciferase reporter vectors, and cultivated for 48 hours before relative luciferase activities were determined (upper panel). HELZ protein expression levels were controlled by immunoblotting (lower panel). (**B**) Renilla luciferase mRNA was measured by RT-qPCR from total RNA extracted in a duplicate experiment presented in (**A)**. Prior to RT, RNA was DNase-treated to digest possibly remaining plasmid DNA. Renilla mRNA levels were normalized to ribosomal protein L28 mRNA levels. (**C**) MCF-7 cells were transfected with the indicated siRNA oligonucleotides and 24 hours later with pRL-SV40, and cultivated for 48 hours before relative luciferase activities were determined (upper panel). HELZ knock-down efficiency was controlled by immunoblotting. Results are presented as mean values±standard error of the mean of n = 3 (**A**) and n = 6 (**C**) independent experiments, respectively.

The identification of HELZ as a novel PABP-interacting protein suggested a role of HELZ in protein translation. Therefore, we co-transfected HeLa cells with V5-tagged HELZ together with a SV40-driven renilla luciferase reporter gene construct. Exogenous overexpression of increasing amounts of HELZ induced relative luciferase activities in a dose-dependent manner (Figure 4A). To investigate whether enhanced luciferase activities were due to upregulation of mRNA levels, we performed RT-qPCR analysis. However, V5-HELZ expression did not influence renilla luciferase mRNA levels (Figure 4B). These effects were promoter-independent as the same regulation was observed with thymidine kinase (TK) and cytomegalovirus (CMV) driven reporter plasmids (data not shown). Vice versa, siRNA-mediated suppression of endogenous HELZ expression reduced luciferase activities (Figure 4C, upper panel). The HELZ siRNA oligonucleotides #9 and #11 efficiently down-regulated HELZ expression as verified by immunoblotting, whereas #12 was ineffective and used as control (Figure 4C, lower panel). Taken together, these data suggest that HELZ positively regulates reporter gene translation rather than transcription or mRNA stability.

**Figure 5:**
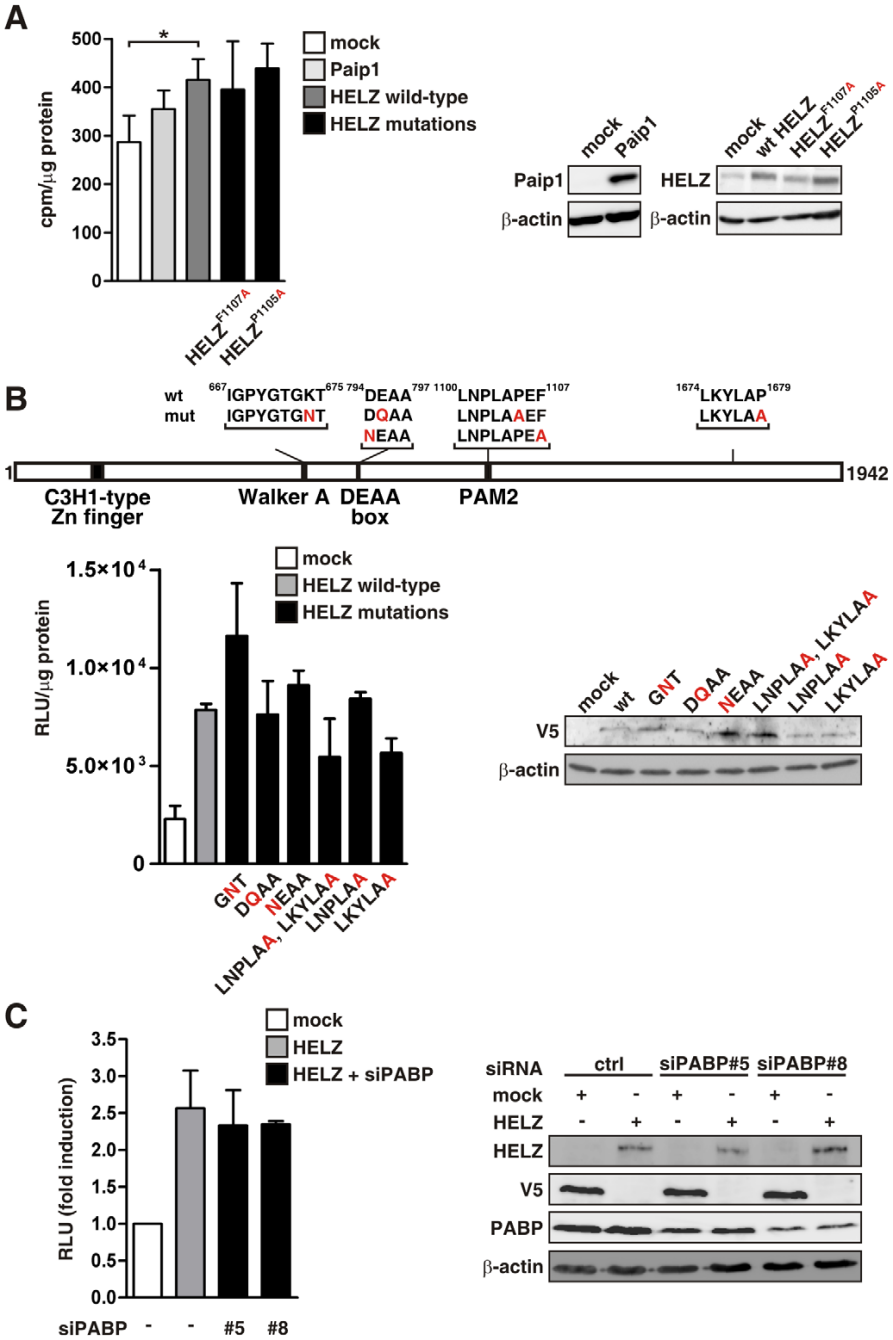
HELZ regulates global protein translation. (**A**) HeLa cells were transfected with 3 µg of the indicated constructs and 48 hours later pulse-labeled with [^35^S]-methionine for 1 hour. Incorporated radioactivity was measured by scintillation counting following TCA precipitation and results were normalized to total protein levels (left panel). Protein overexpression was controlled by immunoblotting (right panel). (**B**) Schematic depiction of the different HELZ mutations (upper panel). HeLa cells were co-transfected with the indicated HELZ constructs together with pRL-SV40 and cultivated for 48 hours before luciferase activities were determined and normalized to total protein concentrations (lower left panel). Expression of V5-tagged constructs was controlled by immunoblotting (lower right panel). (**C**) HeLa cells were transfected with mock control, HELZ or HELZ in combination with PABP siRNA oligonucleotides and analyzed as described under (**B**). Expression of the transfected vectors and PABP knock-down efficiencies were controlled by immunoblotting (right panel). Results are presented as mean values±standard error of the mean of n = 3 independent experiments.

To further investigate the role of HELZ on *in cellulo* protein translation, we performed [^35^S]-methionine incorporation assays. Global translation was significantly induced by exogenous HELZ expression (Figure 5A, left panel), confirming the function of HELZ in protein translation. Surprisingly, mutation of the phenylalanine residue critical for PABP association within the PAM2 motif of HELZ did not influence HELZ-dependent stimulation of translation (Figure 5A, left panel). Expression of the indicated constructs was controlled by immunoblotting (Figure 5A, right panel).

To further elucidate the mechanism of HELZ function in translation we performed additional mutagenesis studies (schematically depicted in Figure 5B). Whereas ATP-binding and ATP hydrolysis motifs have previously been demonstrated to be crucial for eIF4A helicase function [31], mutation of the corresponding residues in HELZ (GNT, DQAA and NEAA) resulted in maintained stimulatory effects on protein translation (Figure 5B, left panel). Moreover, neither proline mutations in the PAM2 motif nor in the C-terminal LxxLAP motif significantly influenced the positive effect of HELZ on translation (Figure 5B, left panel). Exogenous expression of V5-tagged HELZ constructs was controlled by immunobloting (Figure 5B, right panel). In addition, siRNA-mediated suppression of PABP did not influence the stimulatory effect of HELZ on global protein translation, supporting the notion that HELZ function is PABP independent (Figure 5C, left panel). Expression of the transfected vectors and PABP knock-down efficiencies were controlled by immunoblotting (Figure 5C, right panel).

### HELZ promotes cell proliferation and is involved in ribosomal protein S6 phosphorylation

**Figure 6:**
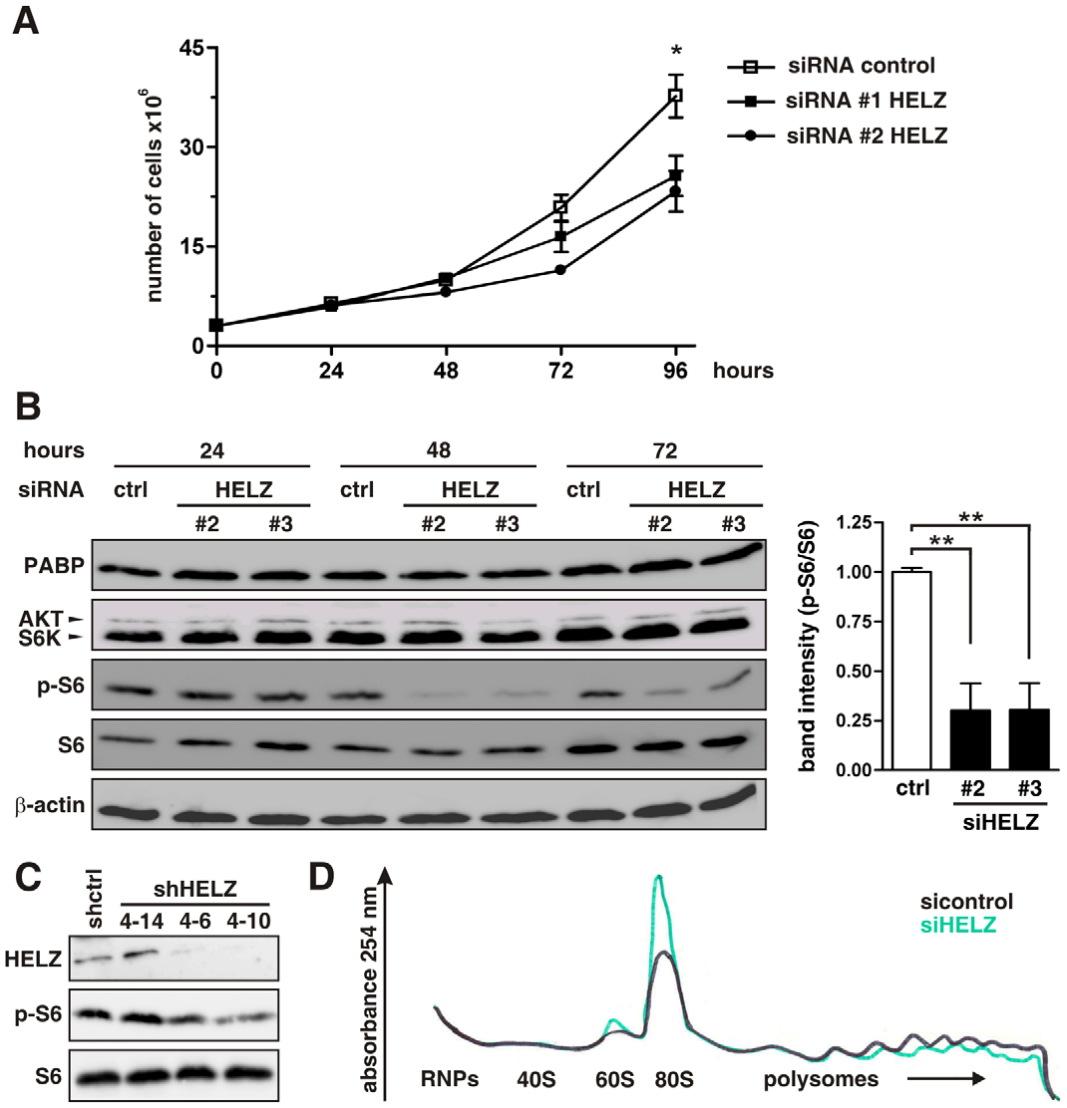
HELZ suppression limits cellular proliferation, ribosomal protein S6 phosphorylation and initiation of translation. (**A**) HeLa cells were transfected with the indicated siRNA oligonucleotides and cell numbers were assessed every 24 hours using an automated cell counter. (**B**) Immunoblot analysis of HeLa cells transfected with two different HELZ siRNA oligonucleotides and harvested at indicated time points after transfection (left panel). The experiment was performed three times independently and the pS6 band intensities were quantified 48 hours after transfection (right panel). (**C**) Protein levels of HELZ, p-S6 and total S6 in stable shRNA control (shctrl) or shRNA HELZ expressing cells were analyzed by immunoblotting. (**D**) Representative polysomal profile (n = 3) of HeLa cells transfected with control or HELZ siRNA oligonucleotides.

Regulation of protein translation impacts cell growth and proliferation. Consistent with a reduction in global protein translation, proliferation of HeLa cells was reduced by siRNA-mediated HELZ suppression (Fig. 6A). Cell viability, as assessed by Trypan blue staining, was not affected in siRNA-silenced cells (data not shown), suggesting that HELZ modulates cell proliferation rather than cell death.

Cellular translation requires a multitude of resources and the mammalian target of rapamycin (mTOR) signaling pathway functions as pivotal integrator [19]. Thus, we analyzed components of the mTOR pathway in HELZ-silenced cells and found a significant downregulation of phosphorylated ribosomal protein S6 (p-S6) compared to control cells (Fig. 6B). Total protein levels of S6 and S6 kinase (S6K), as well as AKT and PABP were not affected. Importantly, stable HELZ downregulation in the HEK293 clone 4–10 (previously described in Fig. 1A) resulted also in attenuation of S6 phosphorylation (Fig. 6C). Clone 4–6 showed a somewhat intermediate HELZ and p-S6 suppression and clone 4–14 served as control, demonstrating dose-dependent attenuation of S6 phosphorylation in an independent tissue culture model.

To determine at which step HELZ is involved in translational regulation, we analyzed polysome profiles from HELZ-silenced and control siRNA oligonucleotides transfected cells. HELZ downregulation resulted in dissociation of polysomes with a concomitant increase in the amount of 80S ribosomes and 60S subunits (Fig. 6D). Consistent with a mainly cytoplasmatic localization, these data suggest that HELZ functions in translational initiation.

## Discussion

Although the specific prolyl residues within the HIFα subunits hydroxylated by PHDs share the sequence LxxLAP, *in vitro* studies on hydroxylase activity using substrate mutations have indicated that very few residues outside the hydroxylated proline itself are critical for hydroxylation [32], [33]. Fragments encompassing the entire HIFα oxygen-dependent degradation (ODD) domain are much better PHD substrates than short 20 amino acid model peptide substrates [34], [35], suggesting that the enzyme-substrate interaction requires multiple contacts. Indeed, crystal structure analysis suggested that the target proline is inserted into the hydroxylase active site while the rest of the unstructured ODD domain adopts an extended conformation around the enzyme [36], [37]. Nevertheless, also non-HIFα proteins have been proposed to be hydroxylated by PHDs within the context of an LxxLAP motif. PHD1 has been reported to hydroxylate Rpb1, the large subunit of the RNA polymerase II, as well as the IκB kinase-β (IKK β), leading to hypoxic activation of NFκB [38], [39]. Our data indicate that conserved LxxLAP motifs can also be found within the PABP-interacting motif PAM2, but the presence of this motif alone cannot be taken as prerequisite for oxygen-dependent regulation. Furthermore, also the only two other proteins harboring two conserved LxxLAP motifs CNOT1 and FGD1, have not been described in the context of oxygen-dependent regulation.

Translational control is a crucial mechanism for a rapid response to physiological changes. Binding of PABP to the poly(A) tail of the mRNA is an important step in translation initiation, since it mediates the circularization of the mRNA through interaction of PABP with eIF4G, a member of the translation initiation complex [40]. Several other PABP binding partners, such as Paip1 and Paip2 have been described to associate through the PABP-interacting motif 2 (PAM2) [28], [41] and modulate PABP function [42], [43]. A bioinformatic survey has identified, among others, HELZ as PAM2 motif containing protein [44] and our data confirm that HELZ interacts with the PAM2 motif with PABP. Surprisingly, mutation of the phenylalanine residue critical for PABP association within the PAM2 motif of HELZ and siRNA-mediated suppression of PABPC1 did not influence HELZ-dependent stimulation of translation, suggesting that the HELZ function in protein translation is independent of PABP. However, we cannot rule out that other cytoplasmic PABPs or inducible PABP (iPABP) might compensate for PABPC1. Interestingly, it has been shown that the PABC1 interactor Tob also associate with iPABP [45] and we can not exclude that HELZ promotes its effect indirectly. Furthermore, PABP-independent regulatory mechanisms of translation initiation are known and HELZ function might be specific for such mRNAs [46]. In addition, the stimulatory function of the putative RNA helicase HELZ on global protein translation was independent of the motifs derived from known RNA helicases, suggesting that either RNA helicase activity is not required for function of HELZ in translation, HELZ is not a RNA helicase or contains additional helicase motifs as previously suggested by Czaplinski *et al.* [5].

Ribosomal protein S6 is located within the 40S small ribosomal subunit and is essential for the translation of mRNAs encoding important components of the translation machinery, such as ribosomal proteins and elongation factors [47]. Phosphorylation of S6 is regulated by mTOR complex 1 (mTORC1) that activates S6 kinase (S6K) as well as compensatory pathways involving AKT or MAPK. Although hypophosphorylation of S6 by HELZ downregulation was independent of total S6K levels, we cannot exclude that HELZ modulated S6K activity directly or alternatively by reducing the activity or expression of a S6K-specific phosphatase. It has been suggested that Type 1 phosphatases can dephosphorylate ribosomal protein S6 and interestingly in this regard, the mammalian alpha4 phosphoprotein, a regulator of the protein phosphatase 2, has recently been described to interact with PABP [48]. Clearly, further experiments are needed to reveal the mechanism by which HELZ promotes S6 phosphorylation.

Human HELZ has alternatively been termed down-regulated in human cancers (DRHC). However, this was based on semi-quantitative RT-PCR analysis of HELZ expression in 95 tumor cell lines and the observation that exogenous HELZ expression inhibited proliferation and colony formation of hepatoma cells *in vitro* [49]. Contrary, the only functional HELZ data published so far suggest an adaptory role for HELZ linking the histone methyltransferase Smyd3 to RNA polymerase II, thereby transactivating oncogenes, homeobox genes and cell cycle regulators [8]. Smyd3 is up-regulated in colorectal and hepatocarcinoma cell lines and Smyd3 overexpression elevated proliferation and colony-formation capacity *in vitro*, suggesting that HELZ functions rather as tumor promoting factor [8]. Although we did not analyse the role of HELZ using tumor formation assays, our findings that HELZ functions as a positive factor in translation support the tumor promoting hypothesis suggested by Hamamoto *et al.*

In summary, we provide evidence that the presence of even two conserved LxxLAP sequence motifs is not indicative of oxygen-dependent proteolytic regulation and describe to our knowledge for the first time a functional role of HELZ in initiation of translation and ribosomal protein S6 phosphorylation.

## Materials and Methods

### Plasmids

Unless otherwise indicated, cloning work was carried out using Gateway technology (Invitrogen, Basel, Switzerland) as described previously [22]. The HELZ^1–1942^ cDNA was obtained from Origene (LabForce, Nunningen, Switzerland) and cloned into pENTR4 (Invitrogen). pDONR-HELZ^1727–1942^ originated from the yeast two-hybrid screen as described previously [22]. Point mutations were generated by site-directed mutagenesis of pENTR4-HELZ^1–1942^ using the QuickChange site-directed mutagenesis kit following the manufacturer's instructions (Stratagene, Agilent Technologies, Basel, Switzerland). The HELZ fragment corresponding to amino acids 1023 to 1199 was amplified by PCR from pENTR4-HELZ^1–1942^. The expression vectors pGEX-6P-1-Paip2^106–127^ and pcDNA3.1-Paip1 were kindly provided by K. Gehring and N. Sonenberg (McGill University, Montreal, Canada), respectively.

### Cell culture and transfections

Human MCF-7 breast carcinoma, human HeLa cervical carcinoma and human embryonic kidney HEK293 cells were cultured in high-glucose Dulbecco's modified Eagle's medium (DMEM) (Sigma, Buchs, Switzerland) as described previously [50], and human hepatoma HuH-7 cells were cultured in RPMI-1640 [51]. For long-term hypoxia, cells were grown in a gas-controlled glove box (InvivO_2_ 400, Ruskinn Technologies, Leeds, UK). Transfections were performed using polyethylenimine (Polysciences, Warrington, PA, USA) as described previously [25] or Lipofectamine2000 (Invitrogen).

### Normoxic and hypoxic mouse tissue samples

Exposure of mice to an inspiratory gas mixture containing 0.1% carbon monoxide for 4 hours resulted in a 50% functional anemia and has been described elsewhere [24].

### Immunoblotting

Immunoblot analyses were performed as previously described [52]. Antibodies used were mouse monoclonal antibody (mAb) anti-HIF-1α (Transduction Laboratories, BD Biosciences, Allschwil, Switzerland), mAb anti-HELZ (Abnova, LucernaChem, Luzern, Switzerland), mAb anti-β-actin (Sigma), rabbit polyclonal antibody (pAb) anti-SP1 (Santa Cruz Biotechnology, LabForce, Nunningen, Switzerland), pAb anti-PABP (Abcam, LucernaChem), pAb anti-AKT, anti-S6K, anti-S6 and anti-pS6 (Cell Signaling, BioConcept, Allschwil, Switzerland).

### mRNA quantification

Total cellular RNA was extracted as described previously [22]. First-strand cDNA synthesis was performed with 1–5 µg total RNA using reverse transcriptase (RT) and mRNA levels were measured by real-time quantitative (q) PCR using internal calibration standards and a SybrGreen qPCR reagent kit (Sigma) in combination with the MX3000P light cycler (Stratagene). To verify RNA integrity and equal input levels, ribosomal protein L28 or S12 mRNA was determined, and the data were expressed as ratios relative to L28 or S12 mRNA levels.

### Immunofluorescence microscopy

Indirect immunofluorescence microscopy was performed as described previously [22]. For synchronization experiments, cells were growth-arrested in the G_0_/G_1_ phase by incubation with 5 µg/ml aphidicolin (Sigma) for 36 hours. Immunofluorescence staining was performed at times 0, 4, 8 and 12 hours after the withdrawal of aphidicolin. Cell cycle stage was determined by staining cells with propidium iodide and analyzed by flow cytometry (BD Biosciences).

### Protein expression and purification

GST and GST fusion proteins were expressed in *Escherichia coli* BL21-AI by induction with 0.025% arabinose for 4 hours and affinity purified using glutathione-sepharose beads (GSTrap FF; GE Healthcare, Dübendorf, Switzerland) according to the manufacturer's instructions.

### Pull-down assays

HeLa cells were grown to 80% confluency and lysed in buffer A (20 mM Hepes/KOH (pH 7.4), 100 mM KCl, 0.1% NP-40, 0.5 mM EDTA, 10% (v/v) glycerol and protease inhibitors (complete EDTA-free, Roche Applied Science, Rotkreuz, Switzerland). Recombinant GST or GST-HELZ fusion proteins (0.5 µg) were incubated with total HeLa cell extracts (2.5 mg) in the presence of glutathione-sepharose beads for 3 hours at 4°C. Beads were washed ten times in buffer A and bound proteins were eluted by incubation in SDS-PAGE loading buffer.

### Transient transfection and luciferase assay

Cells were co-transfected with the indicated firefly luciferase reporter gene plasmids together with 40 ng pRLSV40 renilla luciferase reporter gene vector (Promega, Dübendorf, Switzerland) and luciferase activity was determined using the dual-luciferase reporter assay system according to the manufacturer's instructions (Promega).

### RNA interference

Cells were transfected with either 100 nM siRNA (Invitrogen) or 50 nM siRNA (Dharmacon, Fisher Scientific, Wohlen, Switzerland) duplex oligonucleotides using Lipofectamine 2000 (Invitrogen). The following forward siRNA oligonucleotides (Invitrogen) were used: control siRNA, 5′-GCUCCGGAGAACUACCAGAGUAUUA-3′; HELZ siRNA#2, 5′-UUCAGCUGCAUGCAGCAAAGCACUG-3′; HELZ siRNA#3, 5′-AUGAGGUGGUGUUUACCCAGGACUC-3′. The following forward siRNA oligonucleotides (Dharmacon) were used: HELZ siRNA#09, 5′-CGUGUAUAAAGUCGGGAUA-3′; HELZ siRNA#11, 5′-GCAGUUGAUCCUCGAAUUA-3′; HELZ siRNA#12, 5′-AAUCACAAGCAGCGAGGAA-3′; PABPC1 siRNA #5, 5′-CAUGUAAGGUGGUUUGUGA-3′; PABPC1 siRNA#8, 5′-UGGAUGAGAUGAACGGAAA-3′. The ON-TARGETplus siCONTROL non-targeting pool from Dharmacon was used as control. Expression vectors encoding shRNA sequences targeting HELZ driven by the U6 promoter in a pLKO.1-puro plasmid were purchased from Sigma.

### Metabolic labelling

After transfection of cells with siRNA oligonucleotides or plasmids, cells were incubated for 2 hours in methionine-depleted medium supplemented with 10% (v/v) dialyzed FCS. Cells were pulse-labeled with 20 µCi of [^35^S]-methionine for 1 hour. Finally, cells were washed twice in ice-cold PBS and lysed with buffer containing 10 mM Hepes/KOH (pH 8.0), 1.5 mM MgCl_2_, 10 mM KCl and 0.5% NP-40 supplemented with EDTA-free protease inhibitors for 15 minutes. Lysates were centrifuged for 15 minutes and protein concentrations determined. Total protein (10 µg) was precipitated by 10% trichloroacetic acid, washed twice in ice-cold acetone and radioactivity was measured by scintillation counting.

### Polysomal profiles analysis

HeLa cells were transfected with siRNA oligonucleotides and 48 hours later treated with 100 µg/ml cycloheximide for 15 min at 37°C. Cells were then washed with ice-cold PBS and lysed by addition of 300 µl lysis buffer containing 10 mM Hepes/KOH (pH 7.5), 10 mM MgCl_2_, 50 mM KCl, 2 mM DTT, 0.5% NP-40 and 1% deoxycholate supplemented with EDTA-free protease inhibitors for 15 minutes. Lysates were centrifuged for 15 minutes at 12′000 x *g*. The supernatant was loaded onto a 7–50% sucrose density gradient containing 50 mM Tris-acetate (pH 7.4), 50 mM NH_4_Cl, 12 mM MgCl_2_ and 1 mM DTT. The gradient was centrifuged at 39′000 rpm for 165 minutes at 4°C (SW41 Beckman Coulter) and analyzed at 254 nm using a density gradient fractionator.

### Statistical analysis

Where not otherwise indicated, results are presented as mean values±standard error of the mean of at least n = 3 independent experiments. All statistical tests were performed and graphed using GraphPad Prism v4.0 software (*, *P*<0.05; **, *P*<0.01).

## Supporting Information

Figure S1 Human hepatoma HuH7 cells were growth-arrested in G1 phase by incubation with 5 µg/ml aphidicolin for 36 hours and released from G1 phase by aphidicolin removal. At time points 0, 4, 8 or 12 hours later, cells were fixed with 4% (w/v) paraformaldehyde. Cell cycle progression verified by propidium iodide (PI) staining and FACS analysis (left panel). HELZ subcellular localization was visualized by indirect immunofluorescence and nuclei were stained with Dapi (right panel).
(TIF)

Figure S2 Crude HeLa cell lysates were incubated with recombinant GST, the indicated GST-HELZ fragments, GST-HIF-1α^530–826^ as well as GST-Paip2^106–127^ and GST pull–down was conducted using glutathione–sepharose beads. Eluates were subjected to SDS–PAGE and immunoblotting.
(TIF)

Table S1 **Overview of proteins with two conserved LxxLAP motifs.** Using the PROSITE pattern search tool [53] the Swiss–Prot database was searched for proteins containing two LxxLAP motifs. Conservation analysis across 33 species was subsequently performed using the MULTIZ whole–genome multiple alignment algorithm [54] implemented in the UCSC Genome Browser [55].
(XLS)
