# Supplementary figures and images for: The Putative RNA Helicase HELZ Promotes Cell Proliferation, Translation Initiation and Ribosomal Protein S6 Phosphorylation
Source: PLoS One. 2011 Jul 13;6(7):e22107. doi: 10.1371/journal.pone.0022107 (PMC3135610; doi:10.1371/journal.pone.0022107)

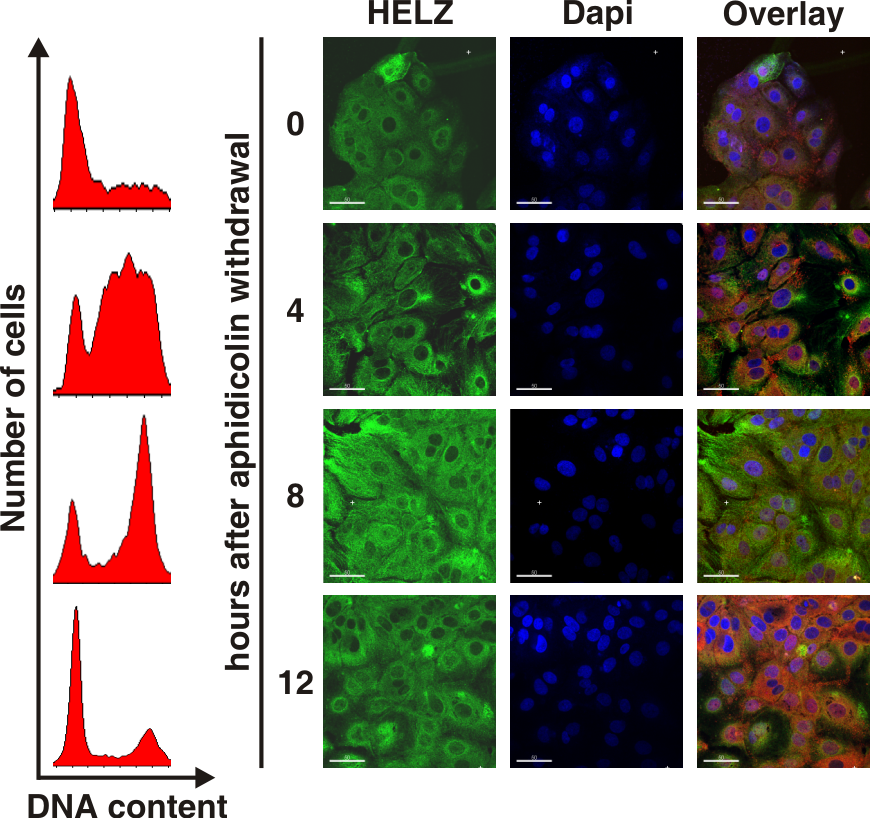

Supplement: Figure S1 — Human hepatoma HuH7 cells were growth-arrested in G1 phase by incubation with 5 µg/ml aphidicolin for 36 hours and released from G1 phase by aphidicolin removal. At time points 0, 4, 8 or 12 hours later, cells were fixed with 4% (w/v) paraformaldehyde. Cell cycle progression verified by propidium iodide (PI) staining and FACS analysis (left panel). HELZ subcellular localization was visualized by indirect immunofluorescence and nuclei were stained with Dapi (right panel). (TIF) [file pone.0022107.s001.tif]

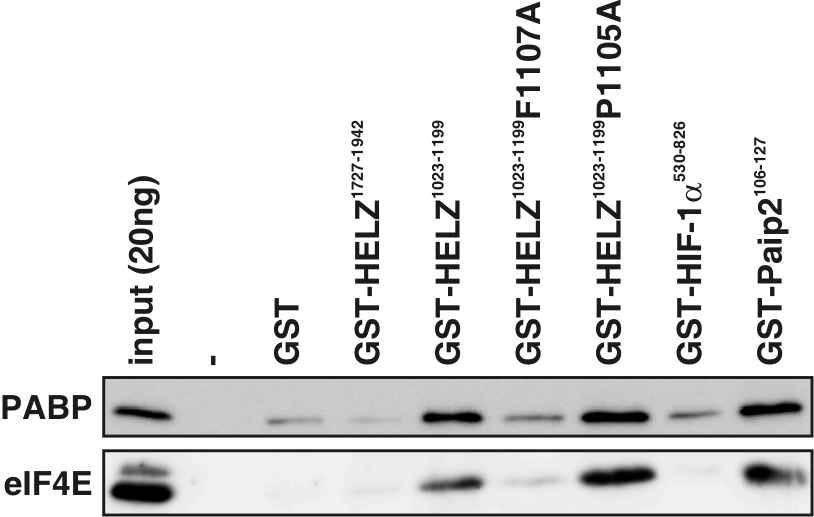

Supplement: Figure S2 — Crude HeLa cell lysates were incubated with recombinant GST, the indicated GST-HELZ fragments, GST-HIF-1α530–826 as well as GST-Paip2106–127 and GST pull–down was conducted using glutathione–sepharose beads. Eluates were subjected to SDS–PAGE and immunoblotting. (TIF) [file pone.0022107.s002.tif]
